# Isoliquiritigenin Enhances the Beige Adipocyte Potential of Adipose-Derived Stem Cells by JNK Inhibition

**DOI:** 10.3390/molecules25235660

**Published:** 2020-12-01

**Authors:** Hanbyeol Moon, Jung-Won Choi, Byeong-Wook Song, Il-Kwon Kim, Soyeon Lim, Seahyoung Lee, Ki-Chul Hwang, Sang Woo Kim

**Affiliations:** 1 Department of Integrated Omics for Biomedical Sciences, Graduate School, Yonsei University, Seoul 03722, Korea; moonstar3636@yonsei.ac.kr; 2 Institute for Bio-Medical Convergence, College of Medicine, Catholic Kwandong University, Gangneung-si 210-701, Korea; gardinia@hanmail.net (J.-W.C.); songbw@ish.ac.kr (B.-W.S.); ilkwonkim@cku.ac.kr (I.-K.K.); slim724@cku.ac.kr (S.L.); sam1017@ish.ac.kr (S.L.); 3 Catholic Kwandong University, International St. Mary’s Hospital, Incheon Metropolitan City 22711, Korea

**Keywords:** adipose-derived stem cells, brown adipocytes, isoliquiritigenin, white adipocytes

## Abstract

Human adipose-derived stem cells (hASCs) can be isolated from fat tissue and have attracted interest for their potential therapeutic applications in metabolic disease. hASCs can be induced to undergo adipogenic differentiation in vitro by exposure to chemical agents or inductive growth factors. We investigated the effects and mechanism of differentiating hASC-derived white adipocytes into functional beige and brown adipocytes with isoliquiritigenin (ILG) treatment. Here, we showed that hASC-derived white adipocytes could promote brown adipogenesis by expressing both uncoupling protein 1 (UCP1) and PR/SET Domain 16 (PRDM16) following low-dose ILG treatments. ILG treatment of white adipocytes enhanced the expression of brown fat-specific markers, while the expression levels of c-Jun N-terminal kinase (JNK) signaling pathway proteins were downregulated. Furthermore, we showed that the inhibition of JNK phosphorylation contributed to white adipocyte differentiation into beige adipocytes, which was validated by the use of SP600125. We identified distinct regulatory effects of ILG dose responses and suggested that low-dose ILG induced the beige adipocyte potential of hASCs via JNK inhibition.

## 1. Introduction

Adipocytes play key roles in energy homeostasis throughout the body and can be classified as white adipocytes (WACs) and brown adipocytes (BACs). WACs are mainly lipid storage cells, whereas BACs are mitochondria-rich multilocular cells that specialize in dissipating energy by generating heat through the action of uncoupling protein 1 (UCP1) [1]. Moreover, beige adipocytes that exist in white adipose tissue (WAT) can be induced under specific conditions such as β3-adrenergic receptor signaling stimulation, exercise, cold exposure, or treatment with small molecules [2]. The beige adipocytes originate from a distinct lineage similar to that of mesenchymal stem cells, which are similar to WACs and BACs, although BACs are derived from Myf5^+^ precursor cells and white and beige adipocytes are derived from Myf5^−^ precursor cells [2,3]. However, there is also evidence that mature white adipocytes can transdifferentiate to beige adipocytes by specific factors [4,5]. There are two types of WAT: subcutaneous and visceral adipose tissues. The adipose tissue under the skin is subcutaneous adipose tissue, whereas the tissue in the abdominal viscera of the mesentery and omentum is visceral adipose tissue [6]. Subcutaneous adipose tissue contains at least two types of adipocytes (mostly white and beige adipocytes). Beige adipocytes express UCP1, which promotes lipid and glucose oxidation, and both brown and beige adipocytes coordinate uncoupled respiration and promote energy expenditure, although these cells develop from separate lineages [7,8].

Brown adipose tissue (BAT) activation and browning of WAT by pharmacological control increase systemic energy consumption. Moreover, unlike genetic engineering, small molecules that induce BAT function likely represent a safer approach to improving overall metabolism. More recently, the literature has suggested that fat browning can be used as a novel therapeutic approach for treating cardiovascular and metabolic diseases [9,10,11]. It has been indicated that epicardial fat functions similarly to BAT and provides heat directly to the cardiac muscle. Thus, epicardial adipose tissue may protect the heart during core body temperature drops or protect the body from adverse hemodynamic conditions such as ischemia and hypoxia [12].

Our research is considered a preclinical application for the treatment of potential cardiovascular diseases through the browning of epicardial adipose tissue (EAT), based on the activation of BAT-specific genes such as UCP1, PRDM16, and PPARGC1A (PGC-1α) in human EAT [13]. These BAT-specific genes will be used as markers of white-to-beige transdifferentiation. Sacks et al. reported that the expression level of UCP-1 in epicardial fat decreased with age and increased with body mass index but appeared to have no relationship to epicardial fat volume. In addition, whether epicardial adipose tissue is BAT or functions as a BAT-like depot is unclear and remains an issue of discussion. UCP1 ablation induced obesity in mice fed a normal diet and vastly augmented high-fat diet-induced obesity in C57Bl6 mice that were exempt from thermal stress by living in a thermogenically non-recruited state [14]. Small molecules promote adipocyte UCP1 expression and improve glucose homeostasis [15,16,17]. A recent study showed that JNK controlled expression of type 2 iodothyronime deiodinase (Dio2), which is required for T4 conversion to T3. It provides evidence that higher UCP1 expression and T3-mediated browning of white adipose tissue by thermogenesis are related [18,19]

Previous studies have proposed promising natural products and therapeutic chemicals, such as flavonoids, for the treatment of obesity and its related complications [20,21,22]. Isoliquiritigenin (ILG, 2′, 4′, 4-trihydroxychalcone) is a flavonoid with a chalcone structure derived from the roots of plants related to licorice, including *Glycyrrhiza uralensis*, *Mongolian glycyrrhiza*, and *Glycyrrhiza glabra*, and it prevents and treats various diseases due to its various pharmacological properties. It has also been considered to have potent anti-inflammatory, antioxidative, anticancer, and cardioprotective properties [23,24,25,26,27,28,29]. A recent paper showed that ILG modulates signaling of the toll-like receptor 4 (TLR4)/myeloid differentiation factor 2 (MD-2) complex at the receptor level [30] and inhibits diet-induced adipose tissue inflammation by strongly inhibiting nucleotide-binding and oligomerization domain (NOD)-like receptor pyrin domain-containing protein 3 (NLRP3) inflammasome activation [31]. ILG suppresses lipid accumulation and inhibits protein-tyrosine phosphatase 1B activation, negatively regulating the insulin signaling cascade in 3T3-L1 cells [32]. ILG also suppresses hepatic steatosis by decreasing fat accumulation by downregulating lipogenic genes and protecting hepatocytes from oxidative damage caused by fat accumulation in high-fat diet-fed mice [33].

Although ILG has various pharmacological activities, the role of ILG in the white-to-beige transdifferentiation of adipose-derived stem cells has never been explored. Moreover, we demonstrated the distinct regulatory mechanisms and functions by which the small molecule ILG dose-dependently induces the white-to-beige transdifferentiation of adipose-derived stem cells.

## 2. Results

### 2.1. The Small Molecule ILG Modulates hASC Adipogenesis

**Figure 1:**
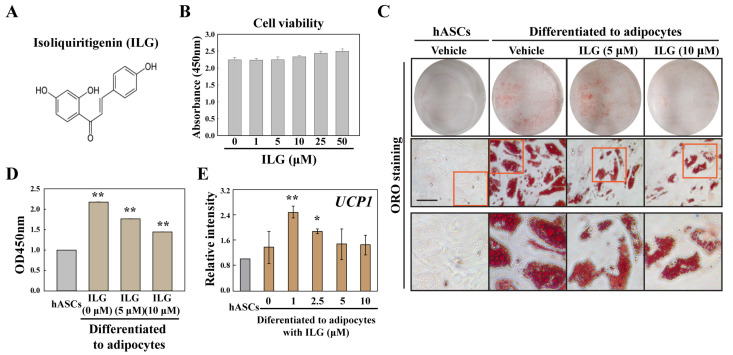
Effects of ILG on the adipocyte differentiation of human adipose-derived stem cells (hASCs). (**A**) Chemical structure of ILG. (**B**) The cell viability of ASCs treated with ILG was determined using the CCK-8 assay. Treatment with the indicated concentrations of ILG for 24 h did not cause any significant change in cell viability compared with that of the control group. (**C**,**D**) Fat droplets were stained with Oil Red O (Scale bar = 200 μm), and accumulated lipids were quantified by measuring the absorbance. (**E**) Gene expression was analyzed by real-time PCR. Expression levels of *UCP1* in low-dose ILG-treated adipocytes differentiated from ASCs. *GAPDH* was used as an internal control to normalize the expression of the target genes; *n* = 3 independent experiments; * *p* < 0.05 and ** *p* < 0.01.

We showed that hASC-derived WACs were able to increase the brown adipogenic potential by low-dose ILG treatment-induced overexpression of UCP1 (Figure 1). ILG has a chalcone structure and was not cytotoxic to hASCs at 0~50 μΜ (Figure 1A,B). We further tested the dose-dependent effects of ILG on lipid droplet formation in hASC-derived WACs, and ILG dose-dependently inhibited lipid droplet biogenesis (Figure 1C,D). Interestingly, although UCP1 expression in WACs was not changed by a high concentration of ILG, a low concentration of ILG induced beige and BAC differentiation with increased UCP1 expression (Figure 1E).

### 2.2. The Small Molecule ILG Induces the Transdifferentiation of White Adipocytes to Beige Adipocytes

**Figure 2:**
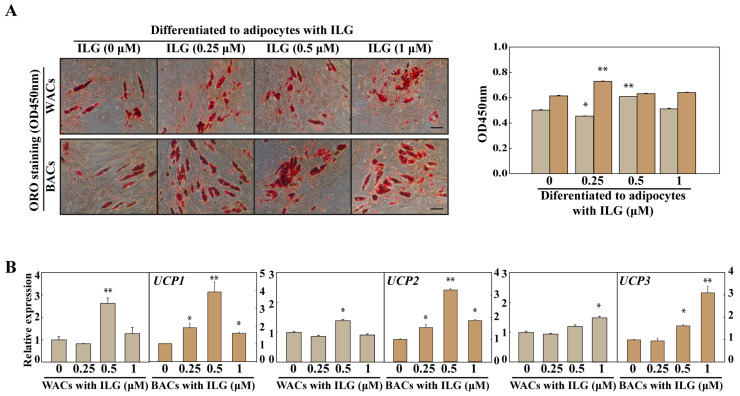
Effects of low-dose ILG on the white-to-beige transdifferentiation of hASCs. (**A**) Representative microscopic images of adipocytes stained with Oil Red O (Scale bar = 200 μm). OD values measured from isopropanol elution of Oil Red O stain after 12 days of differentiation. (**B**) Effect of ILG treatment on the mRNA expression of *UCP* in white adipocytes (WACs) and brown adipocytes (BACs). Gene expression in cells treated with ILG for 24 h after adipogenic differentiation was analyzed by real-time PCR. *GAPDH* was used as an internal control to normalize the expression of the target genes; *n* = 3 independent experiments; * *p* < 0.05 and ** *p* < 0.01.

To investigate the effect of ILG on lipid metabolism, we evaluated the expression of thermogenic pathway markers. As shown in Figure 2, lipid drop quantification by ORO staining showed increased levels in WACs and BACs treated with 0.5 μM and 0.25 μM ILG, respectively. In addition, the expression of UCP1, UCP2, and UCP3 was analyzed to evaluate the activation of the heat-generating pathway and the futile cycle of proton pumping through the activation of *UCPs*. These results showed that ILG significantly increased the expression levels of *UCP1*, *UCP2*, and *UCP3* in both WACs and BACs (Figure 2B).

**Figure 3:**
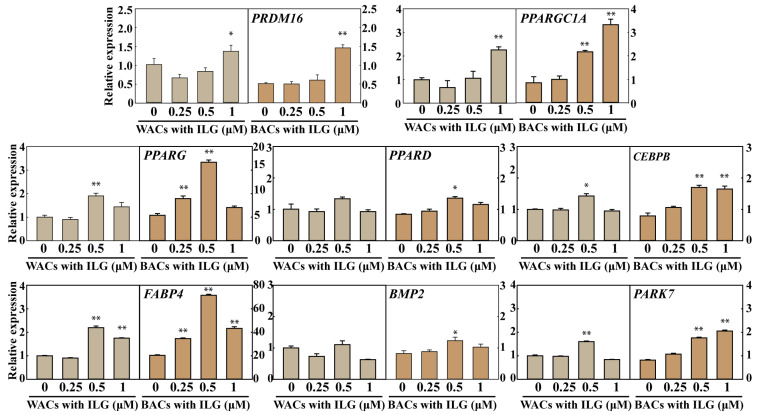
Effect of ILG treatment on the mRNA expression of browning markers in WACs and BACs. Effect of ILG treatment on the mRNA expression of browning markers in WACs and BACs. Quantitative real-time PCR analysis of the *PPARGC1A*, *PPARG*, *PPARD*, *CEBPB*, *FABP4*, *BMP2*, and *PARK7* genes in cells treated with ILG for 24 h after adipogenic differentiation. *GAPDH* was used as an internal control to normalize the expression of the target genes; *n* = 3 independent experiments; * *p* < 0.05 and ** *p* < 0.01.

Moreover, we investigated *PRDM16*, *PPARGC1A*, *PPARG*, *PPARD*, *CEBPB*, *FABP4*, *BMP2*, and *PARK7* expression in WACs and BACs treated with ILG (0, 0.25, 0.5, and 1.0 μM) (Figure 3). We observed significant increases in the mRNA levels of these markers in both WACs and BACs. 

### 2.3. The Small Molecule ILG Induces Beige Adipocyte Differentiation by JNK Inhibition

**Figure 4:**
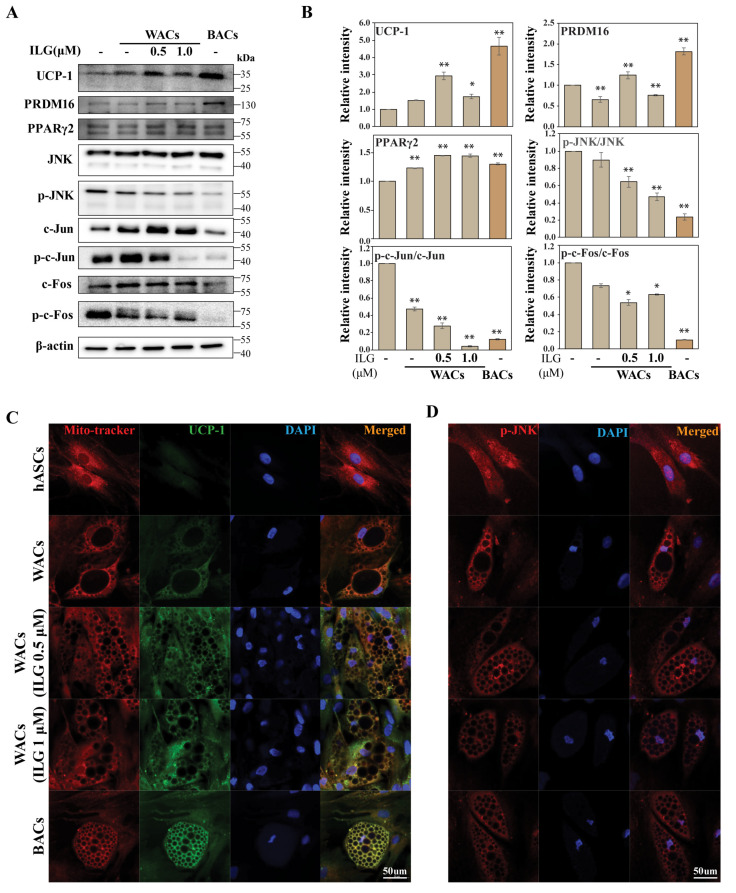
ILG treatment increased the BAC differentiation of WACs. (**A**) Validation of differentially regulated browning markers by immunoblot analysis. (**B**) The expression levels of the indicated BAC-specific proteins were quantified by Western blot analysis. Expression changes of UCP-1 and JNK by ILG treatment. Immunofluorescent staining of (**C**) UCP1 and (**D**) p-JNK. Scale bar = 50 μm. The data were normalized to β-actin antibody and analyzed using ImageJ software; *n* = 3 independent experiments; * *p* < 0.05 and ** *p* < 0.01.

**Figure 5:**
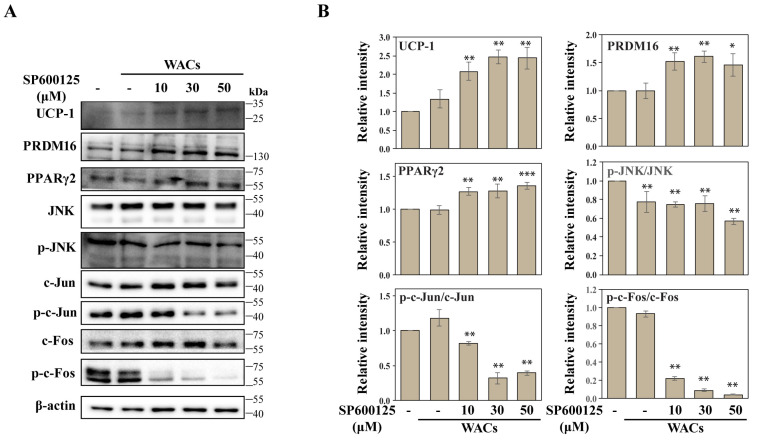
UCP-1 expression was upregulated by JNK inhibition. Cells were treated for 48 h with increasing concentrations (10, 30, and 50 μM) of a JNK inhibitor (SP600125). (**A**) Validation of differentially regulated browning markers and the JNK pathway by immunoblot analysis. (**B**) The expression levels of the indicated BAC-specific proteins were quantified by Western blot analysis. The data were normalized to β-actin antibody and analyzed using ImageJ software; *n* = 3 independent experiments; * *p* < 0.05 and ** *p* < 0.01.

We showed that hASC-derived WACs were able to transdifferentiate to a beige or brown adipocyte by expressing both UCP1 and PRDM16 in response to low-dose ILG treatments (Figure 4A,B). Previously, UCP1 and PRDM16 were shown to be master regulators for brown adipogenesis, and their overexpression could induce browning in stem cells. Our results confirmed that the expression levels of UCP1 and PRDM16 increased in beige and brown adipocytes after ILG treatment of WACs. However, it is thought that WACs contain beige-like adipocytes, because UCP-1 is expressed in WACs even if it is a small amount. Importantly, JNK phosphorylation was downregulated during increasing beige and brown adipocyte differentiation of WACs. Consistently, immunofluorescence staining confirmed a significant increase in UCP1, while p-JNK expression was downregulated in WACs after ILG treatment (Figure 4C,D). To explore the regulatory effect of JNK on beige and brown adipocyte differentiation, a JNK inhibitor (SP600125) was applied to WACs during adipocyte differentiation (Figure 5A,B). Immunoblotting showed that JNK phosphorylation was reduced by the inhibitor, while expression of UCP1 and PRDM16 was dose-dependently increased by the inhibitor.

## 3. Discussion

hASCs are isolated from human lipoaspirate tissue and show functional properties that are of interest in cell therapy and regenerative medicine. Our results revealed that hASC-derived WACs were able to promote brown adipogenesis by expressing both UCP1 and PRDM16 in response to low-dose ILG treatments by inhibiting the JNK signaling pathway.

A large number of studies have reported the beneficial effects of flavonoids on human health, and the bioactive component ILG has been well documented as a potent antioxidant with anti-inflammatory, antiatherosclerotic, cardioprotective, and cancer-preventing properties, but the roles of ILG in stem cell differentiation remain unclear [24,26,34,35]. Moreover, ILG prevents insulin-induced ROS generation in 3T3-L1 cells and suppresses lipid accumulation [32]. Another report showed that ILG (10 μM) treatment had little effect on fat production by differentiated 3T3-L1 cells [36]. Researchers have suggested that ILG can inhibit adipose tissue inflammation in both inflammasome-dependent and inflammasome-independent manners and attenuate adipose tissue fibrosis by targeting innate immune sensors. Recent studies have shown that ILG not only strongly inhibits activation of the NLRP3 inflammasome, but also improves diet-induced adipose tissue inflammation [31]. ILG reduces phosphorylated JNK expression in palmitic acid-induced macrophages, and the induced inflammatory changes were suppressed by inhibiting NFκB activation [36]. In addition, ILG inhibits LXRα-dependent hepatic steatosis through JNK1 inhibition and protects hepatocytes from oxidative injury caused by fat accumulation [33]. Furthermore, other researchers have suggested that ILG induces dose-dependent developmental toxicity and oxidative stress-mediated apoptosis through the Nrf2-HO1/JNK-ERK/mitochondrial pathway in zebrafish embryos and larvae [25]. Similar to our observations, low-dose ILG upregulated the expression of thermogenesis-related genes (UCP1, PRDM16, and SIRT1) in the interscapular BAT of diet-induced obese C57BL/6J mice, increasing insulin sensitivity and energy expenditure and decreasing adiposity [29].

Here, we observed that a low concentration of ILG-induced beige and BAC differentiation with increased UCP1 expression (Figure 1) and increases the expression of UCP1 and PRDM16 and adipogenic differentiation markers in both WACs and BACs in the context of lipid metabolism (Figure 2). Flavonoids have a wide spectrum of biochemical and pharmacological effects and may show variaous responses according to their concentrations. A study has shown that chronic consumption of a blueberry extract was only related to extremely low concentration of anthocyanins in rat brains [37]. Low doses of luteolin increased p21 expression and high doses of luteolin decreased its expression in cancer cells [38]. These results suggest that flavonoid bioactivity does not follow a classical dose-dependent relation and it may have critical biological implications [39]. However, so far, there has been no report to accurately explain the opposite responses and their mechanisms according to the concentration of flavonoids such as ILG.

The JNK signaling pathway has been reported to be involved in the regulation of stem cells’ osteogenic and adipogenic differentiation, and JNK activity is abnormally elevated in obese conditions [40,41,42]. Several studies have shown that JNK is activated in response to ER stress stimulation in high-fat-induced obesity, and these groups also investigated the effect of ER stress on the thermogenic capacity of mouse beige adipocytes [43,44,45,46]. Ucp1 expression is severely suppressed in inguinal WAT under ER stress conditions [43]. Whether JNK is activated after ER stress stimulation and regulates the mRNA levels of *Ucp1* and *Pparγ* in vitro and ex vivo has been investigated [43]. However, the consequences of JNK inhibition on the beige and brown adipocyte differentiation of hASCs are unknown. Therefore, the purpose of this study was to investigate the role of JNK in regulating the differentiation of hASCs to beige and brown adipocytes after ILG treatment. Interestingly, we found that JNK was deactivated by the beige and BAC differentiation of hASCs in response to low-dose (<1 μM) ILG treatments, and the ratio of p-JNK to JNK in WACs dose-dependently declined in response to ILG treatment. We then blocked JNK pathway activation with different concentrations of SP600125 (a specific inhibitor of the JNK pathway). These results indicated that the JNK signaling pathway regulated the beige adipocyte differentiation of hASCs. Consequently, we demonstrated the distinct regulatory functions of the ILG dose responses and induced beige adipocyte potential of hASCs by JNK inhibition in response to low-dose ILG.

## 4. Materials and Methods

### 4.1. Cell Culture, Adipocyte Differentiation, and Reagents

The JNK inhibitor SP600125, insulin, dexamethasone (Dex), indomethacin (Indo), triiodothyronine (T3), rosiglitazone (Rsg), and 3-isobutyl-1-methylxanthine (IBMX) were purchased from Sigma (St. Louis, MO, USA). Human adipose-derived stem cells (hASCs) were purchased from Lonza (subcutaneous fat-derived cells; Walkerville, MD, USA) and cultured in Dulbecco’s modified Eagle’s medium nutrient mixture F-12 (DMEM/F-12, GIBCO, Waltham, MA, USA), supplemented with 10% fetal bovine serum (FBS, Atlas Biological, Fort Collins, CO, USA) at 37 °C for 48 h in a humidified atmosphere of 5% CO_2_. The cells have been cryopreserved at primary passage and express CE13, CD29, CD44, CD73, CD90, CD105, and CD166 and do not express CD14, CD31, and CD45 according to the data sheet. We purchased the hASCs (passage #1), cultured the cells by passage #3, and used cells of the same batch and passage for all experiments. When the cells reached confluence, initial differentiation of the cells was induced by replacing the DMEM/F-12 with differentiation media (WACs and BACs, final conc.: 5 μg/mL insulin, 1 nM T3, 125 μM Indo, 2 μg/mL Dex, 0.5 mM IBMX, and 0.5 μM Rsg) [47]. After 4 days, the cells were switched to maturation media (WACs, final conc.: 10 μg/mL insulin; BACs, final conc.: 5 μg/mL insulin, 1 nM T3, and 1 μM Rsg) for another 6–8 days, and the media were replaced every 48 h. The 10 mM small compound ILG (Tokyo Chemical Industry Co., Ltd., Tokyo, Japan) stock solution was prepared in dimethylsulfoxide (DMSO). WACs and BACs were incubated with different doses (0.25, 0.5 and 1 μM ILG; 10, 30, and 50 μM SP600125) of ILG and SP600125 for 24 h to investigate the effect of ILG and JNK inhibition, respectively. They were analyzed at the expression level using an immunoblot and real-time PCR system.

### 4.2. Cell Viability Assay

To measure the toxicity of ILG, we conducted a cell viability assay (EZ-Cytox; Dogenbio, Seoul, Korea). Cells were seeded on 96-well culture plates at 5 × 10^3^ cells/well, allowed to adhere, and then treated with different concentrations of ILG (1, 5, 10, 25, and 50 μM). After 24 h, to detect cell viability, 10 μL of EZ-Cytox solution was added to each well and incubated at 37 °C for 1 h. Optical density values at 450 nm were measured with a microplate reader (Multiskan FC, Thermo Fisher Scientific, Waltham, MA, USA).

### 4.3. Oil Red O Staining and Measurement of Lipid Content

The hASCs and hASC-derived adipocytes were rinsed with phosphate-buffered saline (PBS), fixed with 4% formaldehyde at room temperature for 1 h and washed again three times with distilled water. An Oil Red O (ORO) solution (Sigma, St. Louis, MO, USA) was layered onto the differentiated cells and incubated for 20 min, followed by three washes with distilled water. Stained adipocytes were observed by light microscopy. Intracellular lipid levels were quantified after extracting the ORO bound to cells with 100% isopropanol (Duksan, Ansan, Korea), and the ORO was measured at 450 nm using a microplate reader (Multiskan FC, Thermo Fisher Scientific, Waltham, MA, USA).

### 4.4. RNA Extraction and Quantitative Real-Time PCR

Total RNA was extracted from cells using an RNA extraction kit (iNtRON Biotechnology, Seongnam, Korea) according to the manufacturer’s instructions, and 1 μg of RNA was converted to cDNA using a Maxime RT PreMix kit (iNtRON Biotechnology). To quantify the expression levels of genes, power SYBR green (SYBR Premix Ex Taq (Tli RNase H Plus, ROX Plus), Takara Bio, Foster City, CA, USA), and a StepOnePlus Real-Time PCR system (Applied Biosystems, Foster City, CA, USA) were used. The primer sequences used for qPCR are listed in Table 1. The transcript levels of each gene were normalized to the level of *GAPDH*, and the relative quantification (2^−∆∆^CT) method was used to analyze the data.

### 4.5. Immunoblot Analysis

Protein extracts were prepared using RIPA buffer (Thermo Fisher Scientific, Waltham, MA, USA) containing 1% phosphatase inhibitor (Thermo Fisher Scientific) and 1% protease inhibitor (Santa Cruz Biotechnology, Paso Robles, CA, USA). For Western blotting, protein extracts were loaded onto SDS-polyacrylamide gels and transferred to a polyvinylidene difluoride (PVDF, EMD Millipore, Burlington, MA, USA) membrane. The membranes were blocked with 5% skim milk in TBS-T and incubated overnight at 4 °C with antibodies against UCP-1 (1:500 dilution; Thermo Fisher Scientific), PPARγ (1:1000 dilution; LifeSpan BioSciences, Inc., Seattle, WA, USA), PRDM16 (Novus Biologicals, Centennial, CO, USA), JNK, phospho-JNK, c-Jun, phospho-c-Jun, c-Fos, phospho-c-Fos (1:1000 dilution; Cell Signaling, Danvers, MA, USA), and β-actin (1:5000 dilution; Santa Cruz Biotechnology, Paso Robles, CA, USA). After being washed, the membranes were incubated for 1 h with secondary antibodies conjugated with horseradish peroxidase (1:2000 dilution; Santa Cruz Biotechnology, Paso Robles, CA, USA).

### 4.6. Immunofluorescence Staining

Cells in 4-well side chambers (SPL, Pocheon, Korea) were fixed with 4% formaldehyde and rinsed with PBS. Permeabilization was performed with 0.1% Triton X-100 (Sigma) for 10 min. After being incubated in blocking solution (2.5% normal horse serum (Vector Laboratories, Burlingame, CA, USA) in PBS) for 1 h, the cells were incubated with rabbit anti-UCP-1 (ThermoFisher Scientific) and p-JNK (Cell Signaling) antibodies (diluted 1:500) overnight at 4 °C. After being washed 3 times with PBS, the cells were incubated with FITC-conjugated anti-rabbit IgG (1:500, EMD Millipore) and rhodamine-conjugated anti-rabbit IgG (1:500, Vector Laboratories) secondary antibodies. DAPI (Invitrogen, Carlsbad, CA, USA) was used to stain the nuclei. Fluorescence images were captured by a confocal microscope (LSM710, Carl Zeiss Microscopy GmbH, Jena, Germany). For mitochondrial staining, MitoTracker Red (1 mM, Invitrogen) was added to the DMEM/F-12 at a concentration of 200 nM for 30 min at 37 °C. After incubation, the cells were washed with PBS, fixed with 4% formaldehyde, washed again with PBS, and immunostained.

### 4.7. Statistical Analysis

All experimental results were compared using one-way analysis of variance (ANOVA) by the Statistical Package of Social Science (SPSS, version 17) program. The data are expressed as the mean ± SEM. A protected least-significant difference (LSD) test, which consists of single-step procedures and one-way ANOVA to analyze multiple comparisons, was used to identify significant differences between the means. A value of *p* < 0.05 was considered statistically significant, and statistical significance was shown as * *p* < 0.05 and ** *p* < 0.01.

## Figures and Tables

**Table 1:** Primer sequences used for quantitative real-time PCR.

Gene	Forward Sequence (5′-3′)	Reverse Sequence (5′-3′)
*UCP1*	GTGTCGGCTCTTATCGCTGG	CCAAGTCGCAAGAAGGAAGG
*UCP2*	CCTCTCCCAATGTTGCTCGT	GGCAAGGGAGGTCATCTGTC
*UCP3*	TCAGCCCCCTCGACTGTAT	CCAGGTTGACCCACGGTAG
*PRDM16*	TGGTTGCCTGCATGAGTGTG	CGGTTAGGAAGACAGCCGAA
*PPARG*	GCAAACCCCTATTCCATGCTG	ACGGAGCTGATCCCAAAGTT
*PPARD*	AGACAGATGCACCAACGAGG	CTGCTCCATGGCTGATCTCC
*PPARGC1A*	TGACCCCGTCTCTCTGAAGT	CTCAGAGTCCTGGTTGCACAT
*CEBPB*	CGACGAGTACAACCGGC	TGCTTGAACAAGTTCCGCAG
*FABP4*	CCTTAGATGGGGGTGTCCTG	AACGTCCCTTGGCTTATGCT
*BMP2*	GGAACGGACATTCGGTCCTT	CACCATGGTCGACCTTTAGGA
*PARK7*	GGTGAGTGGTACCCAACGG	CCTTAATCCCAGCTCGCCTC
